# Supplementary material for: Circulating Cell-Free DNA-Based Liquid Biopsy Markers for the Non-Invasive Prognosis and Monitoring of Metastatic Pancreatic Cancer
Source: Cancers (Basel). 2020 Jul 1;12(7):1754. doi: 10.3390/cancers12071754 (PMC7409337; doi:10.3390/cancers12071754)
Supplement: Supplementary file 1 [file cancers-12-01754-s001.pdf]

cancers

MDPI

Article

Circulating Cell-Free DNA-Based Liquid Biopsy Markers for the Non-Invasive Prognosis and Monitoring of Metastatic Pancreatic Cancer

Marta Toledano-Fonseca, M. Teresa Cano, Elizabeth Inga, Rosa Rodríguez-Alonso, M. Auxiliadora Gómez-España, Silvia Guil-Luna, Rafael Mena-Osuna, Juan R. de la Haba-Rodríguez, Antonio Rodríguez-Ariza and Enrique Aranda

Supplementary Materials:

Supplementary Table 1. Results of baseline RAS mutation analysis in tissue and plasma.

Patient ID	Tissue biopsy RAS status	Liquid biopsy RAS status	Treatment
Patient 1	KRAS Cdn 12	WT	Gemcitabine+Nabpaclitaxel
Patient 2	WT	WT	Gemcitabine+Nabpaclitaxel
Patient 3	N/A1	KRAS Cdn 12	Gemcitabine+Nabpaclitaxel
Patient 4	KRAS Cdn 12	KRAS Cdn 12	Gemcitabine+Nabpaclitaxel
Patient 5	KRAS Cdn 12	KRAS Cdn 12	Gemcitabine+Nabpaclitaxel
Patient 6	KRAS Cdn 12	KRAS Cdn 12	Gemcitabine+Nabpaclitaxel
Patient 7	N/A	KRAS Cdn 12	Gemcitabine+Nabpaclitaxel+FOLFOX
Patient 8	N/A	KRAS Cdn 12	Gemcitabine
Patient 9	WT	WT	Gemcitabine+Nabpaclitaxel
Patient 10	KRAS Cdn 12	KRAS Cdn 12	Gemcitabine+Nabpaclitaxel
Patient 11	KRAS Cdn 12	KRAS Cdn 12	Gemcitabine+Nabpaclitaxel
Patient 12	KRAS Cdn 12	KRAS Cdn 12	Gemcitabine+Nabpaclitaxel+FOLFOX
Patient 13	KRAS Cdn 61	KRAS Cdn 61	Gemcitabine+Nabpaclitaxel
Patient 14	WT	KRAS Cdn 12	Gemcitabine+Nabpaclitaxel
Patient 15	KRAS Cdn 12	WT	Gemcitabine+Nabpaclitaxel
Patient 16	KRAS Cdn 12	KRAS Cdn 12	Gemcitabine+Nabpaclitaxel
Patient 17	N/A	KRAS Cdn 12	Gemcitabine+Nabpaclitaxel
Patient 18	WT	WT	Gemcitabine+Nabpaclitaxel
Patient 19	WT	KRAS Cdn 12	Gemcitabine+Nabpaclitaxel+FOLFOX
Patient 20	WT	WT	Gemcitabine+Nabpaclitaxel
Patient 21	KRAS Cdn 12	KRAS Cdn 12	Gemcitabine+Nabpaclitaxel
Patient 22	N/A	KRAS Cdn 12	No treatment
Patient 23	KRAS Cdn 12	KRAS Cdn 12	Gemcitabine+Nabpaclitaxel
Patient 24	N/A	KRAS Cdn 12	Gemcitabine+Nabpaclitaxel
Patient 25	KRAS Cdn 12	KRAS Cdn 12	Gemcitabine+Nabpaclitaxel
Patient 26	N/A	KRAS Cdn 12 and NRAS Cdn 12	No treatment
Patient 27	KRAS Cdn 12	KRAS Cdn 12 and NRAS Cdn 117	Gemcitabine+Nabpaclitaxel
Patient 28	KRAS Cdn 12	KRAS Cdn 12	Gemcitabine+Nabpaclitaxel
Patient 29	KRAS Cdn 12	KRAS Cdn 12	Gemcitabine+Nabpaclitaxel+FOLFOX
Patient 30	WT	KRAS Cdn 12	Gemcitabine+Nabpaclitaxel
Patient 31	N/A	WT	Gemcitabine+Nabpaclitaxel
Patient 32	WT	WT	Gemcitabine+Nabpaclitaxel
Patient 33	KRAS Cdn 12	KRAS Cdn 12	Gemcitabine+Nabpaclitaxel

Patient 34	KRAS Cdn 12	KRAS Cdn 12	Gemcitabine+Nabpaclitaxel
Patient 35	N/A	KRAS Cdn 12	Gemcitabine
Patient 36	KRAS Cdn 12	WT	FOLFIRINOX
Patient 37	N/A	KRAS Cdn 12	Gemcitabine
Patient 38	N/A	NRAS Cdn 13	FOLFIRINOX
Patient 39	KRAS Cdn 12	WT	Gemcitabine+Nabpaclitaxel
Patient 40	KRAS Cdn 12	KRAS Cdn 12	FOLFIRINOX
Patient 41	KRAS Cdn 12	KRAS Cdn 12	Gemcitabine+Nabpaclitaxel
Patient 42	WT	KRAS Cdn 12	No treatment
Patient 43	KRAS Cdn 12	KRAS Cdn 12	FOLFIRINOX
Patient 44	N/A	KRAS Cdn 12	Gemcitabine+Nabpaclitaxel
Patient 45	KRAS Cdn 12	KRAS Cdn 12	Gemcitabine+Nabpaclitaxel
Patient 46	N/A	KRAS Cdn 12	Gemcitabine+Nabpaclitaxel
Patient 47	KRAS Cdn 12	KRAS Cdn 12	No treatment
Patient 48	KRAS Cdn 12	KRAS Cdn 12	FOLFIRINOX
Patient 49	KRAS Cdn 12	KRAS Cdn 12	FOLFIRINOX
Patient 50	KRAS Cdn 12	KRAS Cdn 12	Gemcitabine+Nabpaclitaxel
Patient 51	N/A	KRAS Cdn 12	Gemcitabine+Nabpaclitaxel
Patient 52	KRAS Cdn 12	KRAS Cdn 12	Gemcitabine+Nabpaclitaxel
Patient 53	KRAS Cdn 13	KRAS Cdn 13	FOLFIRINOX
Patient 54	KRAS Cdn 12	KRAS Cdn 12	FOLFIRINOX
Patient 55	KRAS Cdn 12	KRAS Cdn 12	FOLFIRINOX
Patient 56	N/A	WT	FOLFIRINOX
Patient 57	KRAS Cdn 12	WT	FOLFIRINOX
Patient 58	N/A	KRAS Cdn 12	Gemcitabine+Nabpaclitaxel
Patient 59	WT	WT	Gemcitabine+Nabpaclitaxel
Patient 60	N/A	WT	Gemcitabine+Nabpaclitaxel
Patient 61	N/A	KRAS Cdn 12	Gemcitabine+Nabpaclitaxel

© 2020 by the authors. Submitted for possible open access publication under the terms and conditions of the Creative Commons Attribution (CC BY) license (http://creativecommons.org/licenses/by/4.0/).
